# Ultrasound-Assisted Green Extraction and Hydrogel Encapsulation of Polyphenols from Bean Processing Waste

**DOI:** 10.3390/foods15010030

**Published:** 2025-12-22

**Authors:** Alessandro Bosio, Matteo Beccaria, Vera Lavelli

**Affiliations:** Department of Food, Environmental and Nutritional Sciences (DeFENS), University of Milan, 20133 Milan, Italy

**Keywords:** pod, flavanol, flavonol, ultrasound-assisted extraction, hydrogel, encapsulation

## Abstract

Pod is the major solid waste from bean processing, and its accumulation is expected to increase in the coming years due to the increases in pulse consumption. This study aimed to investigate (a) the efficacy ultrasound (US)-assisted extraction of polyphenols from bean pod; (b) their solubilization rate constants and effective diffusivities (De), which are relevant parameters for scaling-up the process; and (c) the encapsulation efficiencies of the recovered phenolic compounds in hydrogel microbeads. Extracts were obtained either in water with US assistance (50–350 W·L^−1^) at 30 °C; in water at 30, 45 and 60 °C; or in water/ethanol mixtures at room temperature. Extracts were analyzed using HPLC with diode array and fluorometric detectors. The extract from US-assisted extraction, selected as the most promising treatment among those evaluated, was then encapsulated in alginate or alginate/chitosan microbeads. Changes in the solubilization rate constants (from 0.097 to 0.480 min^−1^) and De (from 3.4·10^−11^ to 4.6·10^−9^ m^2^·s^−1^) were observed upon the application of US. Increasing the acoustic energy density was more effective at accelerating solubilization than either increasing the temperature or using ethanol as a cosolvent. Polyphenols were better encapsulated in an alginate/chitosan matrix than in alginate alone, achieving 71% recovery of ferric reducing antioxidant power and 69% recovery of 2,2-diphenyl-1-picrylhydrazyl radical-scavenging activity. The cost-effective alginate/chitosan matrix entrapping of pod polyphenols is potentially useful in various food applications.

## 1. Introduction

Pulses are important meat substitutes due to their high protein content. Moreover, they contain compounds such as polyphenols, phytosterols, dietary fibers and bioactive carbohydrates, which have been associated with various health benefits [1]. Therefore, the per capita consumption of pulses is projected to increase globally over the coming decade, with the largest increase expected in Europe [2]. As a result, the accumulation of processing byproducts from the pulse supply chain is an emerging issue. In particular, pod is the major industrial byproduct, representing approximately 39% of the seed [3]. Previous studies have shown that, similarly to pulse seeds, pulse pods contain various phenolic compounds [3,4,5,6,7,8,9]. The use of phenolic extracts in various food systems is thought to be a sustainable approach due to the antibacterial and antioxidant abilities of phenols, which can increase the shelf life of products by preventing the degradation of their nutritional and sensory properties [10]. Phenolic extracts can be used to replace synthetic additives in foods, which have been linked to safety concerns [10]. There is also an increasing interest in the design of foods fortified with natural antioxidants due to their numerous health benefits [11].

Bean (*Phaseouls vulgaris* L.) pod phenolics have been extracted using methanol [4] or ethanol/water mixtures [3]. Water, which is the most eco-friendly solvent, has been used for phenolic extraction from the seeds of different bean varieties [12], but it has not been applied for extractions from bean pods. For soybean (*Glycine max* L.) pod, phenolics were extracted in water/ethanol mixtures directly [6] or after a preliminary hydrolysis with 1 N HCl [5]. Water has been used for the extraction of green soybean pod [7], pea (*Pisum sativum* L.) and broad bean (*Vicia faba* L.) pods [8]. Comparisons of the phenolic yields obtained with various solvents have demonstrated that the water-soluble fraction of pod phenolics is relevant [7,8].

In general, conventional maceration is used for the extraction of phenolics from pulse pods [4,5,6,8]; however, more effective extraction techniques, such as microwave- or ultrasound (US)-assisted extraction, are emerging [13,14]. Using microwaves heats both water and other dipoles used as solvents, as well as the water inside cells, favoring solvent diffusion in the solid matrix and disrupting the hydrogen bonds holding the target compounds, thereby allowing for their dissolution [13,14]. The application of US induces cavitation due to the formation and implosion microbubbles, which disrupts cell walls, improving mass transfer and the release of intracellular compounds through mechanisms such as acoustic streaming and microjetting [13,14]. In extractions involving vinal (*Neltuma ruscifolia* G.) pod, US-assisted extraction exhibited lower energy consumption than maceration and microwave-assisted extraction [9]. US-assisted extraction (maximum power 200 W, unspecified frequency) with a fixed acoustic energy, i.e., 20% amplitude for 10 min, was proposed for the extraction of phenolics from bean pod [3]. US treatment (maximum power 500 W, 20 kHz) applied at amplitudes from 30 to 50% for 10–20 min to extract phenolics from green soybean pods showed that the maximum yield was obtained when using 50% amplitude for 10.5 min [7]. An extended range of US amplitudes (maximum power not specified, frequency not specified)—from 20 to 100%, applied for 1–15 min—was investigated for the extraction of phenolics from vinal. The experiment showed a non-linear effect, with improved yields at the extremes of the range [9].

To improve the stability and bio-accessibility of phenolic compounds after extraction, encapsulation strategies have been proposed. The encapsulation of antioxidants in hydrogels provides higher encapsulation stability compared to liquid systems due to the diminished oxygen diffusion rate, which is 2.48·10^−9^ m^2^·s^−1^ in water and 1.06·10^−9^ m^2^·s^−1^ in a model viscous oil [15]. Because of this, alginate, which is a natural biopolymer of the uronic acids (mannuronic and guluronic acids) present in brown algae and some microorganisms, has found widespread application both in the food and pharmaceutical industries [16,17,18,19]. Indeed, food processing leads to antioxidants being exposed to heat, light and oxygen, which results in oxidative degradation. Moreover, the release of encapsulated compounds from alginate is dependent on the pH. In low-pH environments, such as in the stomach, the alginate gel network shrinks due to the protonation of free carboxylic groups and decreased repulsive forces between polymer chains, meaning the encapsulated compounds are retained. Conversely, at neutral and alkaline pHs, such as in the intestine, the alginate gel network swells and breaks due to increased repulsive forces; thus, the encapsulated compounds are released [16]. Alginate hydrogels are used for the delivery of different bioactive compounds; however, the alginate gel matrix is porous, which can lead to spontaneous diffusion and loss of small molecular mass compounds [16]. Hence, the use of a complementary polymer, such as chitosan, is a strategy to improve the encapsulation properties of alginate. Chitosan is a natural polysaccharide formed by D-glucosamine and N- acetyl glucosamine units that is derived from the chitin found in the shells of crustaceans [16] and used in various food and pharmaceutical applications [20,21]. Due to its positive charges, chitosan can bind to the negative charges of alginate. Combining these polymers improves not only the encapsulation efficiency but also the mechanical and chemical gel properties, while also favoring thermal resistance [20]. Hence, the combination of alginate and chitosan is of interest in terms of encapsulation and controlled release of bioactive compounds [14,19].

Extensive studies have focused on the upcycling of food waste [22], but there is a lack of research focused on bean pod valorization. Based on the previous studies discussed above, the polar phenolic fraction of pulse pods is relevant [7,8]; meanwhile, US-assisted extraction represents a promising green approach for the recovery of pod phenolics [9]. However, there is a knowledge gap regarding the optimal kinetic parameters for scaling-up the extraction. Therefore, this study proposes a strategy for the extraction of bean pod phenolics using the most eco-friendly solvent (water), as well as their subsequent encapsulation. The specific aims of the present study were (a) to compare the efficacy of US-assisted water extraction with previously proposed procedures; (b) to evaluate various kinetic parameters relevant to industrial scaling-up of the extraction, such as the rate constant of the process and the effective diffusivity of the compounds; and (c) to evaluate the encapsulation efficiency of the recovered pod phenolic fraction in alginate and alginate/chitosan hydrogels.

## 2. Materials and Methods

### 2.1. Plant Materials and Chemicals

Sun-dried pods of the Copafam bean variety (*Phaseouls vulgaris* L.) were obtained from farmers located in the pre-alpine area around Brescia, Northern Italy. All the chemicals and reagents used in the experiments were purchased from Sigma-Aldrich (Milan, Italy).

### 2.2. Phenolic Extraction

Dried pods were milled using a model 32B770 Waring Commercial Blender (Torrington, CT, USA). Different batches of approximately 20 g were processed at high speed for 2 min, and the fraction containing particles with sizes between 0.5 and 1 mm was selected by sieving.

For phenolic extraction, 15 g batches of milled pods were placed inside beakers with 200 mL of distilled water and subjected to US (25 kHz) at amplitudes of 20, 40, 60, or 80% for 2 h using a model VCX 500 US sonicator (maximum power 500 W, Ghiaroni & C, Milan, Italy). During sonication, the temperature was maintained in the range 25–30 °C using an ice bath. The acoustic energy density (AED) was calculated by dividing the actual power dissipated in the beaker (provided by the instrument) by the volume of solution inside. The resulting AED varied in the range 50–350 W·L^−1^. In parallel, phenolic extraction was performed in distilled water without US assistance at 30, 45 and 60 °C for 2 h, or using 80% ethanol in water (*v*/*v*) for 2 h, as previously proposed for bean phenolic extraction [23], or using 40% ethanol in 10 mN HCl (*v*/*v*), as also previously proposed for bean phenolic extraction [24]. Aliquots of the extracts were sampled at different time intervals during extraction. Finally, the total extract was recovered by filtration. Extractions were performed in duplicate by two operators.

### 2.3. Extraction Kinetic Models

The two-sites model was applied according to the following equation (Equation (1)) [25]:

C/Ce = 1 − f·exp(−k_r_·t) − (1 − f)·exp(−k_s_·t) (1)

The first-order (Equation (2)) and second-order (Equation (3)) kinetic models were applied, as described below [26]:

dC/dt = k_1_·(Ce − C); (2)

dC/dt = k_2_·(Ce − C)^2^. (3)

Solving the differential Equations (2) and (3) leads to Equations (4) and (5), respectively [26]:

ln(Ce/(Ce − C) = k_1_·t (4)

C = Ce^2^·k_2_·t/(1 + Ce·k_2_·t). (5)

The diffusion of phenolic compounds from the plant matrix can be described by Fick’s second law [27] as follows:

ln((Ce − C)/Ce) = ln(6/π^2^) − π ^2^·De·t/r^2^ (6)

In the above-reported equations, C (mg·kg^−1^) is the concentration of phenolics at time t; Ce (mg·kg^−1^) is the maximum concentration of phenolics extracted after infinite time; f is a fraction of accessible sites; k_r_ (min^−1^) is rate constant for the rapid desorption phase; k_s_ (min^−1^) is rate constant for the slow diffusion phase; k_1_ (min^−1^) and k_2_ (kg·mg^−1^·min^−1^) represent the pseudo-first order and pseudo-second order rate constants, respectively; De (m^2^·s^−1^) is the effective diffusivity; and r (m) is the average particle radius.

### 2.4. Phenolic Encapsulation in Alginate and Alginate/Chitosan Hydrogel Microbeads

Microbeads were obtained via a vibrating nozzle method using a Buchi B-390 encapsulator (Buchi Italia, Milan, Italy), as described previously [28]. For preparing the inlet solution, sodium alginate was dissolved in water and homogenized using a T 25 Ultraturrax (IKA Werke, Staufen, Germany) before the pod extract obtained via US-assisted extraction (240 W·L^−1^, 30 °C, 30 min) was added. The final concentrations were 1.5% sodium alginate and a 1:4 final dilution of bean pod extract. The hardening solutions were 0.2 M calcium chloride or 0.2 M calcium chloride supplemented with 0.5% of chitosan. The inlet solution was delivered to a 300 μm nozzle using a flow of nitrogen at 0.5 bar. The laminar inlet solution flow was broken by a vibration frequency of 1000 Hz, and the coalescence of microdroplets was prevented using a 1400 V electrostatic field between the nozzle and the hardening solution. The distance between the nozzle and the hardening solution was set at 20 cm. Microbeads were stirred in the hardening solution for 10 min and then filtered through Whatman n. 4 paper filter. The filtrate was recovered and kept at −20 °C for further analysis. The microbeads were washed with water and stored at −20 °C until use. A total of three replicates were run in each condition. For every replicate, 50 mL of the inlet solution, 150 mL of hardening solution and 100 mL of washing solution were used and 42 ± 2 g of microbeads was obtained. Control microbeads made of alginate or alginate/chitosan were produced with no pod extract in the inlet solution.

To assess if the phenolic compounds had been encapsulated, a previously proposed procedure was followed [29]. In brief, 250 mg of alginate and alginate/chitosan microbeads, prepared with or without the pod extract, were mixed with 20 mL of 5% Na-citrate before magnetically stirring for 6 h. Next, the mixtures were centrifuged at 1500 rpm at 25 °C for 15 min. The UV-vis spectra of the supernatants were recorded, and the absorbance was read at 280 nm and 350 nm (corresponding to the maximum absorbance for flavanols and flavonols).

Individual phenolic compounds in the pod extracts and hardening solutions were analyzed via HPLC (as described in Section 2.5). The microbeads could not be dissolved in the eluents used for HPLC analysis of phenolics. Hence, the mass of the encapsulated phenolics was calculated by subtracting the mass of phenolics in the hardening solution (h) from the mass of phenolics in the inlet solution (i), as proposed previously [30,31]. The percentage encapsulation efficiency (%EE) was then calculated according to the following equation:

%EE = 100·(g_phenolics_·L_i_^−1^·0.050 L_i_ − g_phenolics_·L_h_^−1^·0.15 L_h_)/(g_phenolics_·L_i_^−1^·0.050 L_i_) (7)

where 0.050 L_i_ and 0.150 L_h_ are the volumes of inlet and hardening solutions used for each trial, respectively.

The pod extract and microbeads were analyzed for ferric ion reducing antioxidant power (FRAP) and 2,2-diphenyl-1-picrylhydrazyl radical (DPPH) radical scavenging activity, as described in Section 2.6 and Section 2.7, respectively. The percentage recoveries of FRAP and DPPH scavenging activity were calculated as follows:

%FRAP = 100·(μmol Fe(II) eq·g_mb_^−1^·42 g_mb_)/(μmol Fe(II) eq·L_e_^−1^·0.0125 L_e_) (8)

%DPPH = 100·(μmol Trolox eq·g_mb_^−1^·42 g_mb_)/(μmol Trolox eq·L_e_^−1^·0.0125 L_e_) (9)

where 42 g_mb_ is the mass of microbeads obtained for each trial and 0.0125 L_e_ is the volume of pod extract used for each trial (0.05 L of inlet solution contained 0.0125 L of pod extract).

### 2.5. Phenolic Analysis by HPLC

The phenolic contents of pod extracts and hardening solutions were analyzed in duplicate, as described previously [32], using a model Shimadzu LC-20 AD pump coupled to a model Shimadzu SPD-M20A photodiode array detector (DAD) and an RF-20 AXS operated by Labsolution Software vers. 5.54 (Shimadzu, Kyoto, Japan). A 2.6 μm Kinetex C18 column (150 × 4.6 mm; Phenomenex, Bologna, Italy) was used. The column was maintained at 40 °C. The separation was performed by means of a linear gradient elution at a flow rate of 1.5 mL·min^−1^. The eluents were (A) 0.1% H_3_PO_4_, (B) acetonitrile. The gradient was as follows: from 6% B to 20% B in 18 min; from 20% B to 60% B in 7 min; from 60% B to 90% B in 19 min; 90% B for 10 min; and then 6% B for 5 min. DAD analysis was carried out in the 200–600 nm range. Flavonol aglycones were identified using pure standards of quercetin and kaempferol and quantified at 356 nm; quercetin derivatives were identified using rutin, quercetin-3-O-glucoside and quercetin-3-O-glucuronide and quantified with the DAD set at 356 nm. Quantification of unidentified flavonols was performed via a calibration curve built with quercetin-3-O-glucoside with the DAD set at 354 nm. Flavanols were identified using pure standards of catechin and procyanidin B1 and quantified with the fluorometric detector set at λex 230 nm and λem 320 nm. Results are expressed as milligrams of phenolic compound per kilogram of bean pods for the extracts and percent recovery for the beads, as described in Section 2.4.

### 2.6. Ferric Ion Reducing Antioxidant Power (FRAP) Assay

The FRAP assay was performed on both the pod extract and the microbeads, as described previously [33]. Briefly, FRAP reagent was prepared by combining 25 mL of 300 mM acetate buffer, pH 3.6, 2.5 mL of 10 mM 2,4,6-tripyridyl-s-triazine in 40 mM HCl and 2.5 mL of 20 mM FeCl_3_. The reaction mixture contained 0.25–0.5 mg of microbeads or 0.05–0.1 mL of pod extract and 3 mL of FRAP reagent. The mixtures were incubated at 37 °C for 4 min. The increase in absorbance at 593 nm in the clarified mixtures was evaluated against a blank with no sample addition after centrifugation at 1500 rpm at 25 °C for 15 min. For each sample, different dilutions were assessed in duplicate. A methanolic solution of FeSO_4_·7H_2_O was used for calibration. The FRAP values of the microbeads are expressed as percent recovery, as described in Section 2.4.

### 2.7. 2,2-Diphenyl-1-Picrylhydrazyl Radical (DPPH) Scavenging Assay

This assay was performed with both the pod extract and the microbeads, as described previously [34], with some modifications. A 25 mg·L^−1^ solution of DPPH was prepared in methanol. The reaction mixture contained 0.25–0.5 mg of microbeads or 0.05–0.1 mL of pod extract, 0.5 mL of the DPPH solution and 3 mL of methanol. The mixtures were incubated at 25 °C for 60 min (when a constant absorbance value was reached). The decrease in absorbance at 515 nm against methanol was determined in the clarified mixtures after centrifugation at 1500 rpm at 25 °C for 15 min. A methanolic solution of Trolox was used for calibration. DPPH values of the microbeads are expressed as percent recovery as described in Section 2.4.

### 2.8. Statistical Analysis

Statistical analysis was performed using Statgraphic vers. 5.1 (STCC Inc., Rockville, MD, USA). To model the extraction kinetics, the advanced regression function was used. Model performance was evaluated using the coefficient of determination (R^2^), mean absolute error (MAE) and root mean square error (RMSE). To compare samples, ANOVA was employed with the least significant difference (LSD, *p* < 0.05) test to find significant differences among samples. For comparison between the two treatments (alginate and alginate/chitosan as carriers), a two-sample comparison test was used.

## 3. Results and Discussion

### 3.1. Effect of Solvent Type and US-Assisted Extraction on Polyphenol Recovery

**Figure 1:**
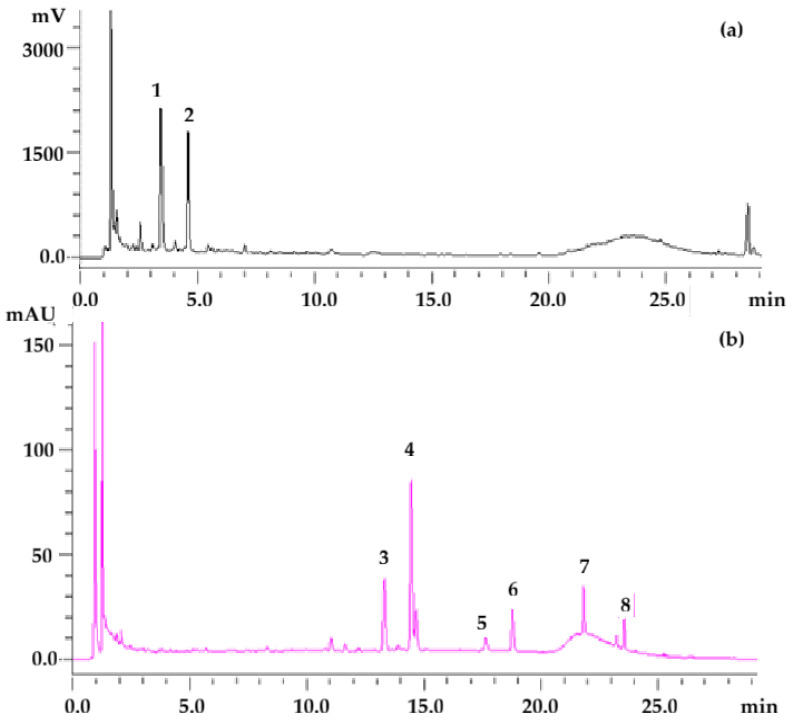
HPLC profile of pod extract in water obtained via US treatment (136 W·L^−1^) for 15 min at 30 °C. (**a**) Fluorometric detector set at λex 230 nm and λem 320 nm; (**b**) DAD detector at 354 nm. 1, procyanidin B1; 2, catechin; 3, rutin; 4, quercetin-3-O-glucoside + quercetin-3-O-glucuronide; 5 and 6, unidentified flavonols; 7, quercetin; 8, kaempferol.

The fluorometric profile of pod extract revealed the presence of catechin and procyanidin B1. The UV-vis profile of pod extracts revealed the presence of rutin, quercetin-3-O-glucoside and quercetin-3-O-glucuronide. Flavonol aglycones, namely quercetin and kaempferol, were also identified. Moreover, two compounds were assigned to flavonols based on UV-vis spectra and may correspond to kaempferol derivatives (Figure 1).

Considering the sum of flavanols and flavonols, water was a better solvent than both 80% ethanol [23] in water and 40% ethanol in 10 mM HCl [24] (Table 1). The latter two solvents were chosen as comparators, because although various solvents have been shown to be useful in extracting phenolics from beans [12], these are the only ones suitable for use as an alternative to water in food processing. The total amount of extracted compounds was 122, 195 and 355 mg·kg^−1^ for 80% ethanol, 40% ethanol in 10 mM HCl and water, respectively. Hence, the water-soluble fraction of bean polyphenols was found to be more relevant compared to the fraction containing molecules that were less polar. Similarly, the water-soluble polyphenols extracted with US assistance from green soybean pod were found to be relevant [7]. Water was also found to be a better solvent than ethanol for pea pods and broad bean pods; however, US was not applied in these extractions [8]. Increasing the water temperature from 30 to 45 °C led to an increase in the total flavanols and flavonols extracted, from 355 to 449 mg·kg^−1^; however, a further increase in temperature to 60 °C was not beneficial for the yield. The use of US resulted in a decrease in extraction time and an increase in yield, which reached 534 mg·kg^−1^ when using US at 240 W·L^−1^ for 30 min or 350 W·L^−1^ for 15 min.

In terms of individual phenolic compounds, US-assisted water extraction at high AED was generally the most effective condition for extracting polar flavanols and flavonol glycosides and glucuronide, while 80% ethanol was the best solvent for extracting flavonol aglycones.

### 3.2. Modeling of Extraction Kinetics

**Figure 2:**
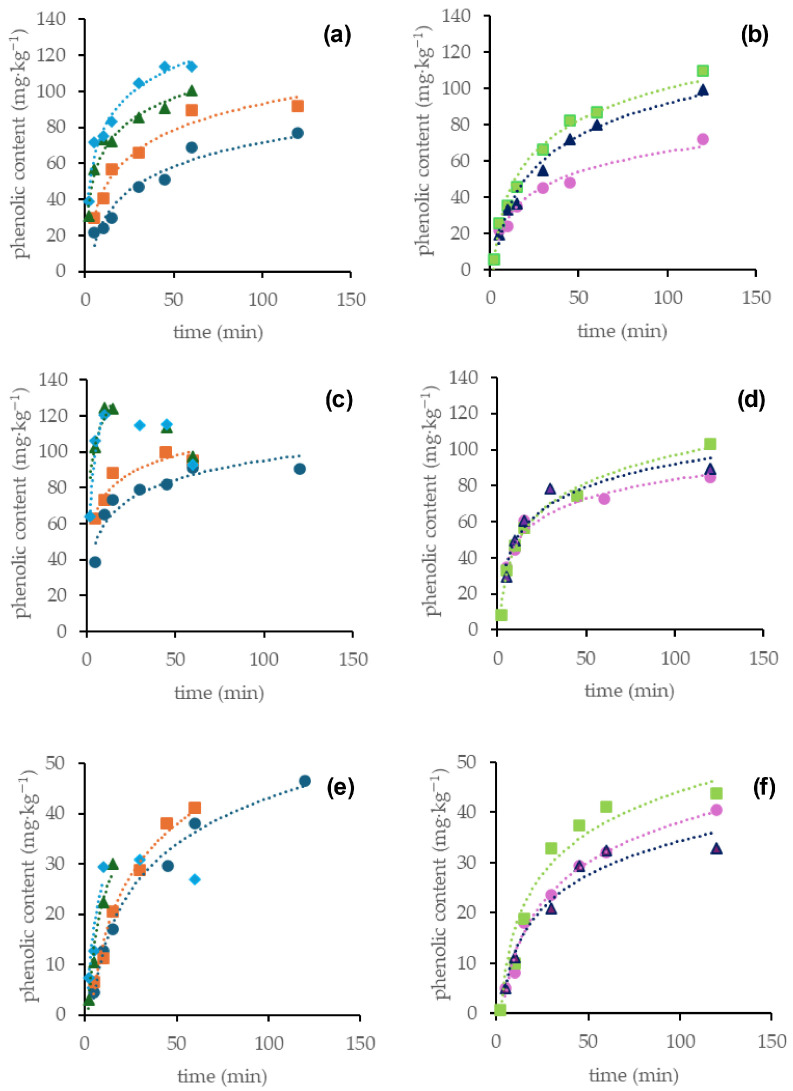
Time course for the water extraction of pod flavonoids: (**a**,**b**) flavanols (procyanidin B1 + catechin); (**c**,**d**) quercetin derivatives (rutin + quercetin-3-O-glucoside + quercetin-3-O-glucuronide); (**e**,**f**) flavonol aglycones (quercetin + kaempferol). Conditions applied were (●): 50 W·L^−1^, 30 °C; (◼): 140 W·L^−1^, 30 °C; (▲): 240 W·L^−1^, 30 °C; (◆): 350 W·L^−1^, 30 °C; (●): 30 °C, (◼): 45 °C; (▲): 60 °C. Each point represents the average of four values.

The time courses of the US-assisted and control polyphenol extractions are shown in Figure 2. For the sake of brevity, the compounds were grouped according to their polarity, based on their HPLC retention times, into flavanols (procyanidin B1 + catechin), quercetin derivatives (rutin + quercetin-3-O-glucoside + quercetin 3-O-glucuronide), unidentified flavonols and flavonol aglycones (quercetin + kaempferol). Increasing AED resulted in an increase in both the initial extraction rate and the total polyphenols extracted. These effects can be attributed to cavitation, which efficiently disrupts cell walls, thereby improving mass transfer and the release of intracellular compounds [13,14]. Previous studies have also observed that US treatment is effective in improving phenolic extraction from green soybean [7] and vinal pods [9]. It also observed that increases in AED can improve US-mediated effects, thus increasing the extraction yield [25]. Moreover, bubble collapse causes high localized temperatures and pressures [13], which could favor the breakage of linkages between phenolics and the cell matrix, also resulting in higher extraction yields. However, prolonged US exposure at a high AED significantly reduced yields, likely due to thermal degradation. Similarly, in other studies it was observed that prolonging US treatment leads to solute degradation [9,35,36].

Polyphenols exhibited varying extraction yields after US treatment (Figure 2). Factors such as differences in polyphenol polarity, which affects binding interactions within the plant matrix and differential responses to US cavitation may have contributed to the observed differences. For instance, for the sum of the polar quercetin derivatives the maximum amount, i.e., 121 mg·kg^−1^ was extracted upon the most intensive US application, i.e., 350 W·L^−1^, 30 °C for 15 min. This amount was much higher than the amount obtained upon conventional maceration in water for 120 min at 30 °C, i.e., 85 mg·kg^−1^ (Figure 2c,d). Conversely, for the non-polar flavonol aglycones, the application of the most intensive US treatment (350 W·L^−1^, 30 °C) for 15 min yielded 32 mg·kg^−1^, while prolonged extraction up to 120 min under the less intensive US application, i.e., 50 W·L^−1^, 30 °C, as well as the conventional maceration at 45 °C led to the maximum extraction of 45 mg·kg^−1^ (Figure 2e,f). It may be argued that the interactions between the non-polar flavonols and the plant matrix were stronger than those between the polar flavonols and the plant matrix and hence they required prolonged US treatment or higher temperature to be broken. The most intensive UV treatment (350 W·L^−1^, 30 °C) was not effective for flavonol aglycones’ extraction because its prolongation led to degradation of these compounds.

It was also noticed that differences in polyphenol structure can lead to differential responses to the effects of US cavitation. In fact, the already described solute degradation occurring for prolonged US treatment at high intensity [9,35,36] was observed for flavonols (Figure 2c,e) but it was not observed for flavanols (Figure 2a).

To gain insights into the extraction kinetics, some models proposed in the literature were applied. First, the two-site model was applied to assess possible heterogeneity in the matrix. Indeed, for heterogeneous matrices, a fraction of the extractable compounds (f) is released easily, with a fast rate constant, and another fraction of the extractable compounds (1-f) is released slowly according to a diffusion-controlled mechanism, with a slow rate constant [25]. In fact, in previous studies on plant matrices such grape pomace, the use of US-assisted extraction revealed the presence of two binding sites for flavonoids [25]. For bean pod polyphenols, the two-site model showed a good fit for both US-assisted and control extraction kinetics, since it depicted high coefficients of determination (R^2^ in the range 95–99.9%). On the other hand, for both US-assisted and conventional polyphenol extraction, f was found to be approximately 1, indicating that the distinction between two binding sites for polyphenols in the pod matrix is not appropriate.

The first- and second-order kinetic models are often used to investigate polyphenol extraction from plant matrices, such as phenolics from onion solid waste [37] and pine needles [38]. These models are used to study diffusion-limited processes that do not display dual-phase extraction due to the absence of the rapid release phase. The first- and second-order kinetic models imply that the extraction rate is proportional to the concentration of soluble components or to the squared concentration of soluble components, respectively. The experimental data obtained in the initial pod phenolics extraction phase (when the trend was increasing) fitted well with the first-order kinetic model considering the R^2^, MAE and RMSE values (Table 2).

Regarding the total phenolics, the first-order rate constants varied from 0.097 to 0.480 min^−1^ when increasing the AED from 0 to 350 W·L^−1^. Other byproducts of plant food processing, which represent potential phenolic sources, have shown slower extraction kinetics. For instance, for the US-assisted extraction of polyphenols from solid onion waste (mean particle size 1 mm) in 90% aqueous glycerol, a first-order rate constant of 0.0206 min^−1^ was observed upon application of 35 W·L^−1^ [37]. On the other hand, some phenolic sources have shown a two-step release behavior, with a very fast release rate in the first step. Indeed, when using an AED of 50 W·L^−1^, the fast rate constant found for US-assisted extraction of grape pomace phenolics (particle sizes in the range 0.425–0.600 mm) in 50% ethanol was 0.260 min^−1^ [25], while the first-order rate constant for pod phenolic extraction upon application of the same AED was 0.118 min^−1^, i.e., slower than the fast rate constant for grape phenolics but faster than the slow rate constant for grape phenolics (0.0290 min^−1^). Obviously, the solubilization rate depends on the particle size of the solid matrix as well as on the AED applied. When the extraction conditions are identical, the extraction rate depends on the individual compounds. The highest first-order rate constants, from 0.098 min^−1^ (30 °C) to 0.38 min^−1^ (350 W·L^−1^ at 30 °C), were observed for quercetin derivatives, followed by flavanols, from 0.036 min^−1^ (30 °C) to 0.25 min^−1^ (350 W·L^−1^ at 30 °C), and flavonol aglycones, from 0.027 min^−1^ (30 °C) to 0.085 min^−1^ (350 W·L^−1^ at 30 °C).

As expected from the time-course extraction trends (Figure 2), the first-order rate constants showed that increasing the temperature from 30 to 45 °C resulted in only a minor increase in extraction rate, while further increasing the temperature to 60 °C caused a decrease. Moreover, increasing the AED was more effective at increasing the rate constant than raising temperature.

Fick’s second law was used to calculate the De values. As expected, increasing the AED led to an increase in De. For the total phenolics, De was 3.4·10^−11^ m^2^·s^−1^ in the control water extraction at 30 °C and 6.3·10^−11^ m^2^·s^−1^ in US-assisted extraction with an AED of 50 W·L^−1^ at 30 °C. In a previous study focused on the recovery of grape pomace phenolics in water, the De was found to be 28·10^−11^ m^2^·s^−1^ when applying an AED of 41 W·L^−1^ [39]. A De equal to 50·10^−11^ m^2^·s^−1^ was observed for the extraction of phenolics from cinnamon bark with 50% ethanol [40]. The De of phenolic compounds can be increased by using deep eutectic solvents. Indeed, the use of choline chloride-based deep eutectic solvents for the extraction of phenolic compounds from cocoa bean shell led to De values in the range 0.98–2.9·10^−11^ m^2^·s^−1^, depending on the solvent formulation [41]. Interestingly, a remarkable increase in De for pod phenolics was observed upon increasing the AED (especially when it was increased to above 140 W·L^−1^). As expected, the De value was also dependent on the nature of phenolic compounds. The De ranged from 4.4·10^−11^ m^2^·s^−1^ (30 °C) to 356·10^−11^ m^2^·s^−1^ (350 W·L^−1^ at 30 °C) for quercetin derivatives; from 2.2·10^−11^ m^2^·s^−1^ (30 °C) to 226·10^−11^ m^2^·s^−1^ (350 W·L^−1^ at 30 °C) for flavanols and from 3.6·10^−11^ m^2^·s^−1^ (30 °C) to 67·10^−11^ m^2^·s^−1^ (350 W·L^−1^ at 30 °C) for flavonol aglycones. The use of US led to a remarkable increase in De, even for the most hydrophobic components (Table 2).

### 3.3. Encapsulation of Polyphenols in Alginate and Alginate/Chitosan Hydrogel Microbeads

The phenolic compounds recovered from bean pod were encapsulated in alginate or alginate/chitosan hydrogels. To assess the encapsulation of phenolic compounds in the microbeads, the samples were treated with 5% Na-citrate, and the UV-vis spectra of the clarified solutions were recorded at the specific maximum wavelengths for flavanols and flavonols, i.e., 280 nm and 356 nm. This approach was proposed in the literature to calculate the efficiency of encapsulation of quercetin in alginate/chitosan microbeads [29]. An increase in absorbance was observed in the alginate and alginate/chitosan microbeads formed in presence of pod extracts relative to the control microbeads gelled in the absence of pod extract, suggesting that encapsulation of flavanols and flavonols had occurred. However, quantification of the encapsulated compounds could not be performed using this approach, since pod extract is a complex mixture in which only a proportion of the compounds have been identified. The use of a complex extract constitutes an important difference from the above-referenced study in which pure quercetin was used [29]. Moreover, the loss of some of the entrapped compounds in the precipitate upon clarification could not be ruled out. Since the microbeads could not be solubilized in the HPLC eluent, the encapsulation efficiency was calculated using the difference between the inlet solution and the hardening solution, as proposed previously [30,31]. Interesting, using this indirect approach, the encapsulation efficiency for pod quercetin in chitosan/alginate (88%) was found to be similar to that found for pure quercetin using the direct quantification of microbeads (82–87%, depending on the chitosan content) [29].

Encapsulation of polyphenols in alginate [42] or alginate/chitosan [43,44] was expected, since previous attenuated total reflection Fourier transform infrared (ATR-FTIR) studies showed that gelation of these hydrocolloids in the presence of polyphenols results in spectral shifts and the appearance of new peaks in the polymeric network, indicating the that phenolics can be wrapped in the matrix and interact with the biopolymers. Different encapsulation efficiencies were observed for the hydrogel systems and were dependent on the polarity and mobility of the compounds involved (Table 3).

For catechin, which is a highly hydrophilic relatively low molecular mass compound, the efficiency of encapsulation in alginate was approximately 30%, which is similar to that found previously [45]. The main reason for the low encapsulation efficiency of low molecular mass polar compounds is diffusion from the droplet to the hardening solution prior to gelation, rather than loss from hydrogel particles during hardening [30]. Indeed, phenolics have higher diffusion rates through liquid–liquid environments before gelling occurs (droplets–hardening solution) than through gel–liquid (microbeads–hardening solution) environments [30]. However, the flavanol dimer procyanidin B1, which has approximately double the molecular mass of catechin, was encapsulated at 65% efficiency. Quercetin derivatives, including the glycosides and glucuronide, exhibited lower encapsulation efficiencies (56%) than quercetin aglycone (79%), despite their higher molecular mass. This difference can be attributed to the higher polarities of quercetin derivatives compared to the aglycone, which favors their diffusion in the hardening solution. Overall, the retention of total pod phenolics in alginate was 53%, while for grape skin phenolics the encapsulation efficiency in alginate was 68% [46]. However, the presence of the copolymer chitosan increased the encapsulation efficiency for the most hydrophobic compounds, namely the aglycones quercetin and kaempferol, whose encapsulation efficiency changed from 79 and 65% to 88 and 87%, and improved the total recovery from 53 to 64% (Table 3).

Besides the indirect evaluation of encapsulation efficiency of individual phenolic compounds, a direct evaluation of the antioxidant activity of the alginate and alginate/chitosan microbeads was performed using the FRAP and DPPH assays. For pod extract, the recovery of the reducing power, i.e., FRAP values, from the hydrogel systems increased from 67% for the alginate matrix to 71% for the alginate/chitosan matrix (Table 3). Moreover, the recovery of DPPH scavenging activity increased from 63 to 69% in the presence of chitosan (Table 3). The electrostatic bonds between the negative anionic groups of alginate and the positive groups of chitosan underlie the formation of a stronger hydrogel from the binary mixture than from alginate alone [29,31]. Hence, this binary mixture was applied to increase the encapsulation efficiency above that expected with pure alginate [28,29,31]. It is worth noting, however, that besides chitosan, other biocompatible and biodegradable polysaccharides or proteins are being considered as copolymers for alginate, including fibers from unconventional sources such as carrob and cocoa [47], which could further improve encapsulation efficiency. For instance, in a similar approach aimed at the encapsulation of phenolic compounds from dandelion leaf extract, the retained antioxidant capacity, determined as the ability to scavenge the ABTS (2,2-azino-bis(3-ethylbenzothiazoline-6-sulfonic acid) radical was 61% in plain alginate and 81% in alginate supplemented with a copolymer, i.e., whey protein [35].

The binary alginate/chitosan system is well established as a strategy to deliver drugs [15,48]. Additionally, it was found that the alginate/chitosan system allows for controlled release of a bioactive phenolic compound, namely quercetin, over 24 h under simulated intestinal conditions [29]. Comparing the release ability of alginate to that of an alginate/chitosan network, it was observed that the influence of chitosan was relatively low [31]. For alginate alone or combined with chitosan, polyphenols are released via the same mechanism at pH 6.8, i.e., diffusion and erosion of the polymeric network [31].

Besides the delivery of encapsulated compounds in vivo, the alginate/chitosan network is also exploited for its functionality in foods or edible films. For instance, in previous studies, pure quercetin and dihydromyricetin were encapsulated in an alginate/chitosan system to develop a matrix exhibiting antibacterial and antioxidant activities [44,49]. The results of the current study show that alginate/chitosan matrices can also be functionalized with cost-effective extracts, such as food processing byproducts (bean pod), to provide antioxidant properties that are potentially useful in various food applications.

## 4. Conclusions and Future Prospects

US-assisted green extraction of pod polyphenols in water was investigated. Considering the total phenolic compounds, increasing the AED was more efficient for accelerating the solubilization process than either increasing the temperature or using ethanol as a cosolvent without US treatment. The kinetic study indicated that the solubilization of phenolics was a diffusion-limited process without a rapid initial phase and followed first-order kinetics. The use of Fick’s second law allowed for the calculation of the De for total phenolics, monomeric and dimeric flavanols, quercetin derivatives and flavonol aglycones; the De values were similar to those of well-exploited phenolic sources, i.e., grape pomace, cinnamon bark and cocoa shell. Based on the kinetic parameters provided here, eco-friendly pod phenolic extraction (using water) can be scaled up, with the AED adjusted according to the desired processing time. On the other hand, the less polar flavonol aglycones were better extracted in ethanol/water mixtures than in water. As polyphenolics are a complex family of compounds, further studies should investigate the optimal extraction conditions for the apolar fraction of bean bod phenolics, as well as the compounds that could be released due to hydrolysis. For this purpose, applying the experimental design could allow for the systematic evaluation the relevant factors and support the development of pod applications.

The extract from US-assisted extraction, selected as the most promising treatment among those evaluated, was then encapsulated in alginate or alginate/chitosan hydrogel microbeads, which are polymers widely used in different food applications involving bioactive compounds. A good recovery of the antioxidant activity of the pod extract was observed when using the microbeads, especially in the presence of the copolymer chitosan. This encapsulation strategy could be further improved with the use of other polymers or technologies targeted to a particular food application.

In conclusion, in the current study, in view of the expected increase in pulse production, a protocol for recovering the water-soluble phenolics from the major solid byproduct of bean processing was developed. The encapsulation yield obtained can be optimized in future pod polyphenol recovery strategies. Ultimately, these data are instrumental for performing techno-economic analyses and predicting the operating conditions for industrial implementation.

## Figures and Tables

**Table 1:** Concentrations of soluble phenolic compounds in dry bean pod using different solvents and extraction conditions *.

	Phenolic Compounds (mg·kg^−1^)								
Solvent and Extraction Conditions	P-B1	C	R	Q-G	u-F1	u-F2	Q	K	Sum
80% Ethanol in water, 25 °C, 120 min	5.0 ^a^	4.6 ^a^	6.6 ^a^	19 ^a^	7.6 ^a^	23 ^a^	25 ^d^	32 ^e^	122 ^a^
40% Ethanol in 10 mN HCl, 25 °C, 120 min	9.7 ^b^	23 ^c^	26 ^b^	62 ^bc^	7.3 ^a^	28 ^a^	20 ^c^	17 ^ab^	195 ^b^
Water, 30 °C, 120 min	62 ^e^	9.9 ^b^	26 ^b^	59 ^b^	45 ^b^	112 ^b^	15 ^b^	25 ^d^	355 ^c^
Water, 45 °C, 120 min	79 ^g^	30 ^d^	32 ^cd^	71 ^cd^	61 ^c^	132 ^c^	18 ^bc^	27 ^d^	449 ^f^
Water, 60 °C, 120 min	66 ^f^	33 ^e^	25 ^b^	65 ^bc^	57 ^c^	107 ^b^	11 ^a^	21 ^c^	384 ^d^
Water, 50 W·L^−1^, 30 °C, 60 min	47 ^c^	22 ^c^	31 ^c^	68 ^cd^	57 ^c^	137 ^c^	15 ^b^	20 ^bc^	399 ^e^
Water, 140 W·L^−1^, 30 °C, 45 min	69 ^f^	22 ^c^	31 ^c^	72 ^d^	59 ^c^	161 ^d^	15 ^b^	23 ^cd^	450 ^f^
Water, 240 W·L^−1^, 30 °C, 30 min	54 ^d^	29 ^d^	35 ^d^	80 ^e^	81 ^d^	222 ^e^	16 ^b^	17 ^ab^	534 ^g^
Water, 350 W·L^−1^, 30 °C, 15 min	51 ^d^	32 ^de^	39 ^e^	82 ^e^	81 ^d^	220 ^e^	16 ^b^	16 ^a^	537 ^e^
Pooled SE	1	1	1	2	2	3	1	1	2

* P-B1, procyanidin B1; C, catechin; R, rutin; Q-G, quercetin-3-O-glucoside + quercetin-3-O-glucuronide; u-F1 and u-F2, unidentified flavonols; Q, quercetin; K, kaempferol. SE, standard error. Data are the average of four values. Different letters in the same column indicate statistically significant differences (LSD; *p* < 0.05).

**Table 2:** Kinetic parameters obtained by fitting the first-order kinetic model and the Fick’s second law model to the water extraction of pod phenolics under different conditions *.

	Ce (mg·kg^−1^)	k_1_ (min^−1^)	R^2^	MAE (mg·kg^−1^)	RMSE (mg·kg^−1^)	De·10^11^ (m^2^·s^−1^)	R
Total							
30 °C	317	0.097	91	18	24	3.4	0.88
45 °C	425	0.105	97	23	39	4.8	0.91
60 °C	394	0.094	99	5	8	2.7	0.93
US, W·L^−1^ 50, 30 °C	358	0.118	92	26	25	6.3	0.95
US, W·L^−1^ 140, 30 °C	410	0.148	88	30	31	12	0.96
US, W·L^−1^ 240, 30 °C	548	0.248	99	4	5	232	0.94
US, W·L^−1^ 350, 30 °C	546	0.480	97	4	7	460	0.97
Flavanols							
30 °C	69	0.036	91	5.1	8.6	2.2	0.95
45 °C	105	0.049	98	4.3	9.2	3.9	0.99
60 °C	99	0.03	97	3.6	5.0	3.5	0.99
US, W·L^−1^ 50, 30 °C	77	0.033	93	3.8	6.1	4.1	0.98
US, W·L^−1^ 140, 30 °C	90	0.069	96	3.8	5.9	18	0.99
US, W·L^−1^ 240, 30 °C	95	0.15	87	7.3	9.7	70	0.90
US, W·L^−1^ 350, 30 °C	106	0.25	85	8.8	11	226	0.98
Quercetin derivatives							
30 °C	73	0.098	93	4.0	5.5	4.4	0.91
45 °C	95	0.103	97	6.9	21	4.3	0.92
60 °C	88	0.08	99	1.0	1.3	4.3	0.85
US, W·L^−1^ 50, 30 °C	87	0.13	95	3.4	5.7	8.3	0.91
US, W·L^−1^ 140, 30 °C	95	0.18	89	3.6	4.5	81	0.94
US, W·L^−1^ 240, 30 °C	123	0.34	97	7.7	23	293	0.97
US, W·L^−1^ 350, 30 °C	123	0.38	99	7.3	22	356	0.95
Flavonol aglycones							
30 °C	52	0.027	98	1.7	2.8	3.6	0.98
45 °C	57	0.038	94	3.1	2.0	10	0.99
60 °C	43	0.037	98	1.5	5.2	2.3	0.99
US, W·L^−1^ 50, 30 °C	61	0.026	98	2.1	3.0	4.1	0.98
US, W·L^−1^ 140, 30 °C	63	0.031	99	2.6	5.4	9.6	0.98
US, W·L^−1^ 240, 30 °C	52	0.085	98	3.1	7.9	46	0.99
US, W·L^−1^ 350, 30 °C	67	0.085	95	13.0	36	67	0.89

* Ce, equilibrium concentration; k_1_, first-order rate constant; R^2^, coefficient of determination; MAE, mean absolute error; RMSE, root mean square error; De, effective diffusivity; R, coefficient of correlation.

**Table 3:** Encapsulation efficiency for polyphenols and the recovery of FRAP values and DPPH scavenging activity of bean pod water extracts in alginate or alginate/chitosan microbeads *.

	Encapsulation Efficiency (%)	
	Alginate	Alginate/Chitosan
C	35 ^aA^ ± 6	32 ^aA^ ± 2
P-B1	65 ^bcA^ ± 4	66 ^bcA^ ± 1
R	56 ^bA^ ± 1	59 ^bA^ ± 2
Q-G	56 ^bA^ ± 3	69 ^cA^ ± 4
u-F1	64 ^bcA^ ± 8	72 ^cdA^ ± 8
u-F2	66 ^cA^ ± 1	89 ^eB^ ± 4
Q	79 ^dA^ ± 2	88 ^eB^ ± 2
K	65 ^bcA^ ± 6	87 ^eB^ ± 3
sum	53 ^A^ ± 1	64 ^B^ ± 3
FRAP	67 ^A^ ± 1	71 ^B^ ± 1
DPPH	63 ^A^ ± 1	69 ^B^ ± 1

* P-B1, procyanidin B1; C, catechin; R, rutin; Q-G, quercetin-3-O-glucoside + quercetin-3-O-glucuronide; u-F1 and u-F2, unidentified flavonols; Q, quercetin; K, kaempferol. Data are expressed as the average ± SE. Different capital letters in the same row indicate statistically significant differences due to the matrix (LSD; *p* < 0.05); different lowercase letters in the same column indicate statistically significant differences due to the compound (LSD; *p* < 0.05).

## Data Availability

The original contributions presented in this study are included in the article. Further inquiries can be directed to the corresponding author.

## Abbreviations

The following abbreviations for chemical compounds and analyses are used in this manuscript:	
P-B1	procyanidin B1
C	catechin
K	kaempferol
Q	quercetin
Q-G	quercetin-3-O-glucoside + quercetin-3-O-glucuronide
R	rutin
u-F1 and u-F2	unidentified flavonols
DPPH	2,2-diphenyl-1-picrylhydrazyl radical
FRAP	Ferric Ion Reducing Antioxidant Power
The following abbreviations for physical processes and relative modeling are used in this manuscript:	
AED	acoustic energy density
De	effective diffusivity
US	ultrasound
C (mg·kg^−1^)	concentration of phenolics at time t
Ce (mg·kg^−1^)	maximum concentration of phenolics extracted after infinite time
f	fraction of accessible sites
k_r_ (min^−1^)	rate constant for the rapid desorption phase
k_s_ (min^−1^)	rate constant for the slow diffusion phase
k_1_ (min^−1^)	pseudo-first order rate constant
k_2_ (kg·mg^−1^·min^−1^)	pseudo-second order rate constant
r (m)	average particle radius.
The following abbreviations for statistical analysis are used in this manuscript:	
LSD	least significant difference
MAE	mean absolute error
RMSE	root mean square error
R	coefficient of correlation
R^2^	coefficient of determination
SE	standard error
